# Recurrent Nicolau syndrome associated with subcutaneous glatiramer acetate injection—a case report

**DOI:** 10.1186/s12883-015-0504-0

**Published:** 2015-12-02

**Authors:** Chiara Zecca, Carlo Mainetti, Roland Blum, Claudio Gobbi

**Affiliations:** Multiple Sclerosis Center, Neurocenter of Southern Switzerland, Ospedale Regionale, Lugano, 6903 Switzerland; Department of Dermatology, Ospedale Regionale Bellinzona e Valli, Bellinzona, 6500 Switzerland; Department of Dermatology, Inselspital, University Hospital, University of Bern, Bern, 3010 Switzerland

**Keywords:** Glatiramer acetate, Multiple sclerosis, Nicolau syndrome, Skin reactions

## Abstract

**Background:**

Glatiramer acetate is worldwide used as first line treatment in relapsing remitting multiple sclerosis. Local skin reactions associated with glatiramer acetate are common, however, only isolated cases of severe local injection site reactions known as Nicolau Syndrome have been reported so far.

**Case presentation:**

We describe the case of a recurrent Nicolau Syndrome occurred during longstanding glatiramer acetate treatment in a woman with multiple sclerosis. The haemorrhagic patch necrotized and was treated locally as a deep second degree burn with excision of dead skin tissue and was healed. Treatment with glatiramer acetate was definitely suspended.

**Conclusions:**

GA injections can be complicated by isolated or recurrent Nicolau Syndrome, a potentially life-threatening condition of which neurologists should be aware.

## Background

Glatiramer acetate (GA) [1] is worldwide one of the most frequently prescribed immune modulatory treatment for multiple sclerosis (MS) and considered both an efficacious and safe compound [2, 3]. GA may cause common albeit generally unserious local skin reactions. These may have potentially negative impact on patient’s health-related quality of life [4] and reduce compliance and adherence, therefore representing a major limitation of GA use [5]. The most common GA related skin reactions include local redness, erythema, swelling, pain, pruritus, bruising, irritation, inflammation, induration of the skin around an injection site, or lipoatrophy, developing in 7–90 % of treated patients [3, 4].

Only single cases of a severe local injection site reaction known as Nicolau Syndrome (NS) have been reported during GA treatment so far [6–12]. The typical NS presentation is pain around the injection site soon after injection followed by erythema developing within hours with a livid discoloration at the injection site. Irregular lightning-like extensions therefore develop peripherally. The epidermis is intact during the first days and an indurated infiltrate is palpable at the injection site. After several days a central necrosis of the size corresponding to the indurated infiltrate develops. After several weeks, a well-demarcated necrotic area remains.

We report the case of a recurrent Nicolau Syndrome (NS) occurring during GA treatment.

## Case presentation

The patient is a 58 year-old female with relapsing remitting MS treated with subcutaneous GA since 2006, and otherwise unremarkable medical history. She presented at our department with a painful livedoid and haemorrhagic skin lesion that developed on her left abdomen approximately 24 h after GA injection. She reported correct injection practice.

She had no fever. Blood cell count, C-reactive protein, renal function, creatine kinase (CK), IgE, autoimmune screening, lupus anticoagulants and cryoglobulins were unremarkable. Superficial ultrasonography at skin lesion showed diffuse oedema without fluid collections. The patient was dismissed with symptomatic non-steroidal anti-inflammatory treatment and GA was stopped.

**Fig. 1:**
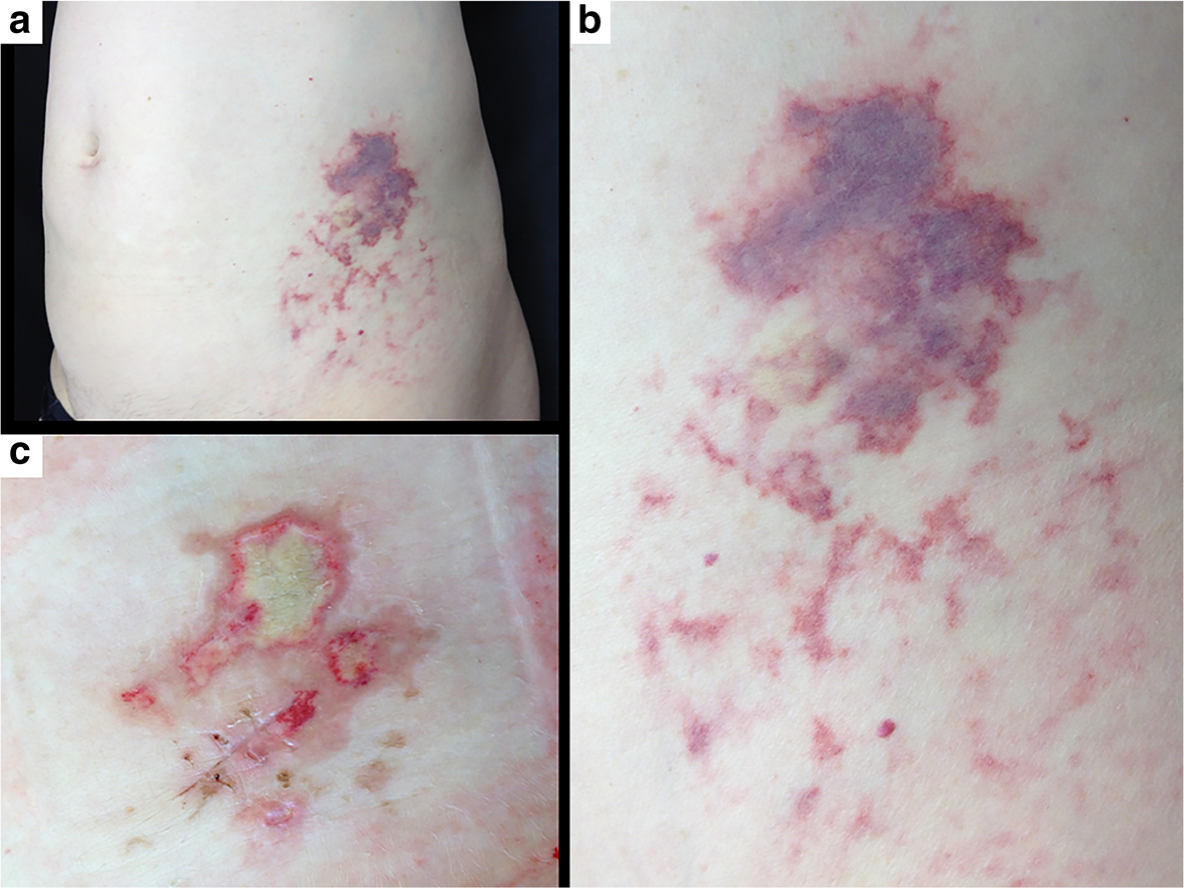
**a** Nicolau Syndrome (NS) on left abdomen at first dermatological evaluation 2 days after onset. **b** Magnification of the NS lesion highlighting an erythematous, purpuric and haemorrhagic patch, at the site of the subcutaneous glatiramer acetate (GA) injection. Surrounding livedoid reticular patch as sign of vascular damage. **c** Three weeks after GA injection the livedoid patch disappeared. A skin necrosis induced by the cutaneous ischemia an atonic superficial wound developed thereafter

**Fig. 2:**
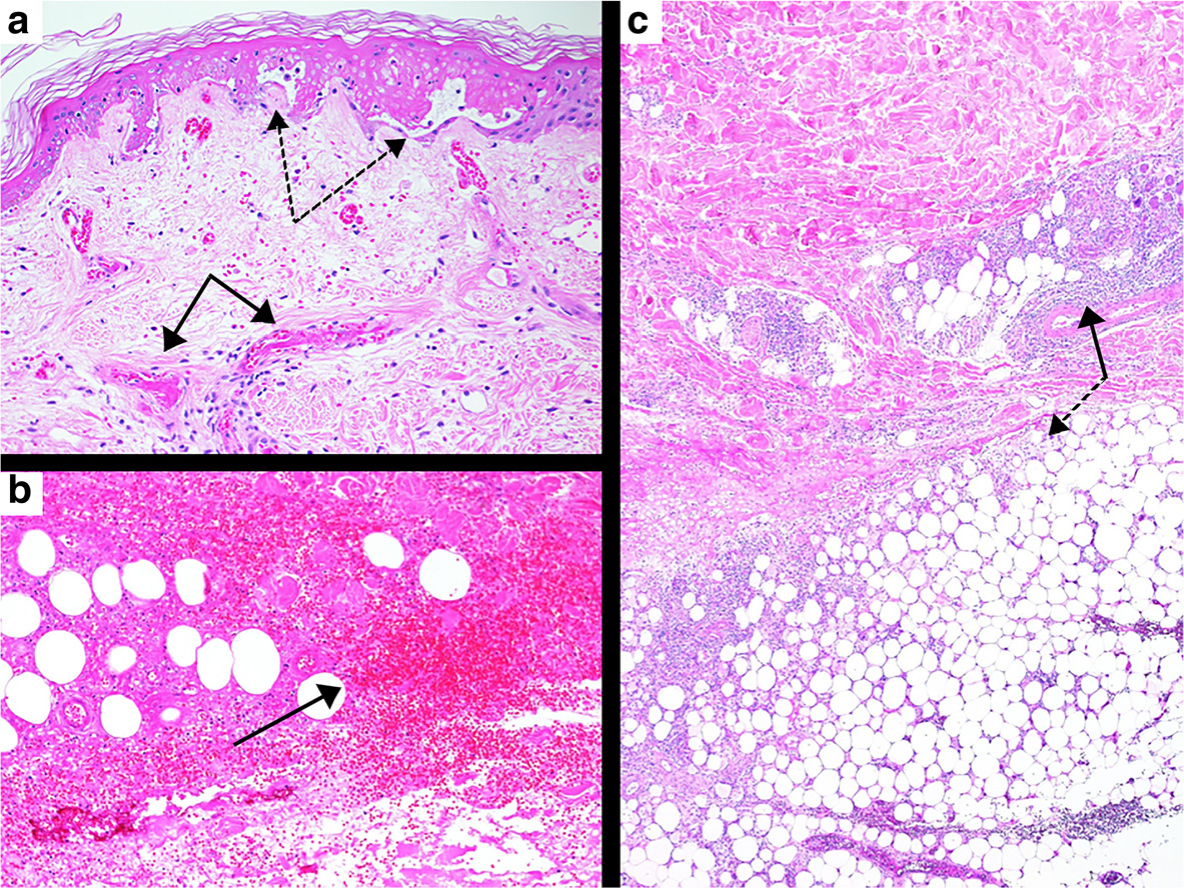
Histological picture of the patient’s skin biopsy. **a** partly necrotic and elevated epidermis (*dashed arrows*) due to thrombosized small vessels (*solid arrows*) in the upper dermis with only sparse cellular inflammation. **b** haematoma in the deeper part of dermis (*solid arrow*). **c** cellular inflammation in the deeper dermis (*solid arrow*) and panniculitis (*dashed arrow*) with coagulative necrosis of dermal collagen and local adipose tissue. Stain: haematoxylin-eosin, a: x 200, b: x 100, c: x 40. Software: ProgRes CapturePro v2.8.8

After 2 days, a livedoid violaceous skin patch with dendritic extensions below the injection site became apparent (Fig. 1a and b). At surgical evaluation at the Emergency Department cellulitis was suspected and a surgical revision proposed to avoid potentially lethal complications (such as sepsis and renal failure following rhabdomyolysis). Considering the negative inflammatory parameters and normal renal and CK values, we referred her before surgery to the Dermatology department for further counselling. Here, a NS was suspected and confirmed by a skin biopsy showing coagulative necrosis of dermal collagen and local adipose tissue, as well as several hyaline thrombi inside small and medium-sized blood vessels (Fig. 2), without skin infection (cellulitis). Bacteriological culture of lesion samples grew only resident flora. The livedoid figures around the central haemorrhagic skin lesions disappeared spontaneously after a week. The haemorrhagic patch of NS necrotized (Fig. 1c). The skin necrosis was limited at the dermis and was removed by a surgical debridement. It was treated with a continuing multifunctional dressing to cleanse, fill, absorb and moisten the wound. A healing by second intention was obtained in 2 months after first evaluation. GA was definitively suspended. At that time, the patient reported that in 2007 a similar lesion had occurred after GA injection in her right abdomen. She experienced immediate and unusual local pain with subsequent appearance of a dark red area around the injection site. A central dark lesion developed within 2 days leading her to the emergency department. The lesion was initially treated conservatively, however, a surgical excision was required after a few days. No histological examination was performed at that time. The lesion evolved into a slightly depressed white scar. A recurrent NS was therefore diagnosed.

## Conclusions

We describe the case of recurrent NS under GA treatment in a patient with MS.

NS (or *Embolia cutis medicamentosa*) is a very rare and potentially life threatening complication following injection of several drugs [13]. It consists of a local vasculitis which can evolve in subcutaneous, fat or muscle tissue necrosis. NS is mostly described as a complication of a variety of intra-muscular drug preparations, however it has also been reported to occur with sc. injections, particularly GA and interferons [14]. Typically, NS onset is represented by pain around the injection site, which is followed by erythema progressively evolving in a livedoid and then haemorrhagic patch. Finally, aseptic necrosis of skin, subcutaneous fat, and muscle tissue can occur [13].

The cause of NS seems to be related to the injection itself rather than to the injected compound or even the administration route (i.e. intramuscular or subcutaneous). Its pathogenesis is not fully understood, and only hypothesis have been proposed so far. An unintended intra-periarterial or perinervous injection might induce severe pain and consequently sympathetic nerve mediated vasospasm. This, in turn, may lead to local necrosis. Injection itself might also cause embolic occlusion of small skin arteries, as well as marked arterial wall inflammation ultimately causing tissue necrosis.

NS is per se rare and only few cases have been previously reported after GA injection so far [7–12]. Notably, only one of these was recurrent [9]. Predominantly females were involved as in our case probably reflecting MS epidemiology. Lesion site was heterogeneous among cases (abdomen, tight, arm, buttock), and NS occurred generally years after GA treatment initiation. No specific trigger factors were identified, and the injection technique was mostly correct with the exception of a single patient referring difficulties with the injection preceding NS onset [8]. In two cases no specific treatment was performed, one resolved completely [10] and the second one evolved in a slightly depressed scar [11]. The remaining patients where treated with surgical excision of the necrotic area, vasodilatative drugs, heparine, fusidic acid and steroids [7, 8, 11, 12] with variable results ranging from no sequelae [8] to muscle involvement [6].

Various treatments have been used for NS, however, none of them has become standard of care. In the majority of cases treatment of NS remains conservative, with analgesics, antibiotics, and dressing. If tissue necrosis is present, surgical removal is performed first, and advanced dressings according to the extent, depth and severity of the wound is therefore used. In wide lesions skin grafts or flaps may be required [15].

It has been suggested that the occurrence of a NS does not contraindicate continuation of GA treatment, as NS is believed to be mainly a consequence of the injection technique rather than of the drug itself [9]. We were not able, however, to identify incorrect injection practice in our patient who nevertheless experienced a recurrence of NS, possibly suggesting individual predisposition and/or a role of the specific compound.

Neurologists should be aware of NS as possible consequence of GA injection, as its course might include lethal complications such as sepsis and renal failure following rhabdomyolysis.

## Consent

Written informed consent was obtained from the patient for publication of this Case report and any accompanying images. A copy of the written consent is available for review by the Editor of this journal.

## Abbreviations

CK: Creatine kinase
GA: Glatiramer acetate
IgE: Immunoglobulin E
MS: Multiple sclerosis
NS: Nicolau Syndrome
